# Auricular Acupuncture Associated with Reduced Waist Circumference in Overweight Women-A Randomized Controlled Trial

**DOI:** 10.1155/2019/6471560

**Published:** 2019-12-18

**Authors:** Felicity Lillingston, Paul Fields, Randall Waechter

**Affiliations:** ^1^Windward Islands Research & Education Foundation, True Blue, Grenada; ^2^School of Medicine, St. George's University, True Blue, Grenada; ^3^Office of Research, St. George's University, True Blue, Grenada

## Abstract

Obesity and subsequent ill health have reached epidemic proportions in developed countries, and many developing countries are on the same trajectory. Weight loss and sustaining a healthy weight have posed a significant challenge for individuals, patients, health-care providers, and public health experts. The literature suggests that dietary advice and lifestyle changes alone have limited sustainable impact for those who are seeking to achieve a healthy weight. Supplementary techniques to control weight, such as acupuncture and auricular acupuncture (AA), have shown mixed results and failed to clearly demonstrate a conclusive impact. This study aimed to provide clarity about the impact of AA on weight loss via a randomized controlled trial. Data were collected from patients to identify measurable girth reduction, weight loss, dietary choices, and mood changes over seven weekly sessions of AA (*n* = 30) versus sham needle as control (*n* = 28). Results demonstrated a large and highly significant AA treatment effect for reduced waist circumference over the course of the seven-week intervention. While the treatment effect for weight loss and BMI was not significant, this negative result may have been mediated by the relatively short duration of the study. Results also demonstrated a significant mood improvement across participants in both the AA intervention and control group as the intervention progressed. Further studies are required to determine if the reduction in waist circumference is driven specifically by the AA alone or in conjunction with improved mood. The results also have potentially significant implications for healthcare delivery in the fight against overweight and obesity.

## 1. Introduction

Overweight and obesity has become a major public health epidemic currently affecting 1.9 billion adults aged 18+ worldwide and resulting in 2.8 million deaths annually [1]. Being overweight and obese increases the risk of serious and potentially life-shortening conditions including type 2 diabetes, heart disease, stroke, and certain cancers [2]. Given the reach of the epidemic and its connection to chronic disease, obesity has been classed a chronic relapsing progressive disease [3] requiring immediate action to control and prevent its global impact. Despite this, assisting patients in achieving and maintaining a healthy weight poses a constant challenge for health-care providers. To date, there is little evidence of a positive long-term effect of any one weight loss intervention, and the number of people with obesity-related disease continues to increase [4]. The reasons for this are complex, including causal factors such as economic growth, an increase in readily available high calorie, low fat addictive foods, and a sedentary lifestyle [5]. Processed foods containing refined carbohydrates, sugars, and refined fats are addictive [5], readily available [6], and inexpensive [7], making them a preferred food choice for many people, and the food industry has taken advantage of this addictive trend [8]. These highly refined, fatty, sugary foods demonstrate an addictive potential [9] and trigger a dopamine response in the reward center of the brain [10–12] that can diminish over time, requiring increased intake of the sugars to experience the same release of dopamine and subsequent reward [11–13]. The potential is for a vicious cycle of increased sugary food consumption, increased calorie intake, and increasing weight [14]. The use of acupuncture was noted as early as 1972 to assist patients with opioid drug addiction and is still used today [15, 16]. In 1996, the WHO listed drug abuse as a medical problem that could be helped by acupuncture [17]. Previous successes using auricular acupuncture (AA) to help patients with addictions to alcohol, drugs, and smoking provide an impetus to expect a similar result when using AA for a sugar addiction [18]. We surmised that intervening in this addictive pathway may help individuals reduce and control their weight. To examine this, some of the addiction points demonstrated by NADA [19] to work for detox and addictions to drugs and alcohol were manipulated. The aim was to determine if AA can reduce craving for sweet refined foods, leading to a reduction in weight and body mass index (BMI) among overweight women. To date, there is little evidence of the effectiveness of AA on weight loss. Existing trials are at best descriptive of short duration, and the designs fell short of sound scientific rigour [20, 21]. There is an urgent need for well-designed and implemented studies to examine the effectiveness of AA for weight loss [22]. This study attempts to address some of these shortcomings by following a strict randomized controlled trial design.

## 2. Methods

A randomized controlled trial was conducted to examine the hypothesis that AA could contribute to reducing weight, waist circumference, and BMI compared with a sham AA treatment.

A total of 60 participants were assigned at random to receive an AA (*n* = 30) or sham AA procedure (*n* = 30) once a week for seven weeks. The same researcher, who was a qualified acupuncturist, administered the treatment or placebo to all participants. The study was single-blind, in that the participants did not know their treatment group, but the researcher administering the treatment was aware of the group assignment for each participant. The study was carried out on the campus at St. George's University in Grenada, West Indies. The participants were primarily medical and veterinary students attending the university. Strict inclusion/exclusion criteria were followed. All participants were women between the ages of 20–30 with a BMI of 25 or higher. Women in this age group were only selected for the trial as recent research has demonstrated that a patient's age and gender can significantly impact weight loss [23]. A systematic review by Robertson and colleagues found evidence that men and women respond differently to weight loss [24], and it was noted during weight management programs for both men and women that losing weight may be more difficult for women, partially due to metabolic differences between genders [25, 26]. Therefore, the present study focused on this potentially higher-risk group.

Participants were recruited by flyers that were posted in prominent locations on the university campus. The trial was open to female staff, students, and significant others who met the stated inclusion/exclusion criteria.

At the first appointment, the medical history was reviewed for each study candidate and any potential concerns that could be impacted by study involvement were flagged. Participants were also asked if they had any allergies—for example to nickel or white metals—and whether they had a previous needle shock issue, though needle shock or vasovagal responses are uncommon in acupuncture, i.e., 0% to 7% [27].

During the study, two participants were withdrawn from the trial. One participant started a new medication midway through the study that had a side effect of decreasing appetite. The other participant stopped eating midtrial due to relationship issues. Both participants were in the placebo group and their data were not used for formulating the trial results. However, both participants wished to continue to come for treatments as they felt the time spent each week to be beneficial. No one chose to drop out of the trial during the study. At the end of the trial, the final sample sizes were AA group *n* = 30 and placebo/sham group *n* = 28.

### 2.1. Inclusion and Exclusion Criteria for the Trial

Strict inclusion and exclusion criteria were defined and implemented as previous studies lack credibility due to poor research design [21, 22]. Age and gender can affect weight loss [22–26], so these factors were taken into consideration when designing the trial. Inclusion criteria included females between 20 and 30 years of age, with a BMI of over 25.0, as this is seen by NHLBI [3] as the categorization threshold for overweight or obese. Exclusion criteria were any participant with a BMI of under 24.9 (i.e., under normal range). Participants were asked to disclose any medical conditions they were being treated for, and those with any metabolic disorder that could affect weight were also excluded from the trial. Participants were also asked to disclose any prescribed medications. Routine medications were acceptable as long as the participant had been taking it for 3 + months and would be continuing on the same regime throughout the trial. If a new medication was started or stopped for whatever reason, the participant was asked to notify the trial coordinator and the participant would subsequently be excluded from the trial. A consent form was signed by each participant to this affect.

### 2.2. Ethical Clearance

The study received ethical clearance by the Institutional Review Board (IRB) at St. George's University in January 2017. The SGU IRB is registered with the US Department of Health and Human Services.

## 3. Materials

**Figure 1:**
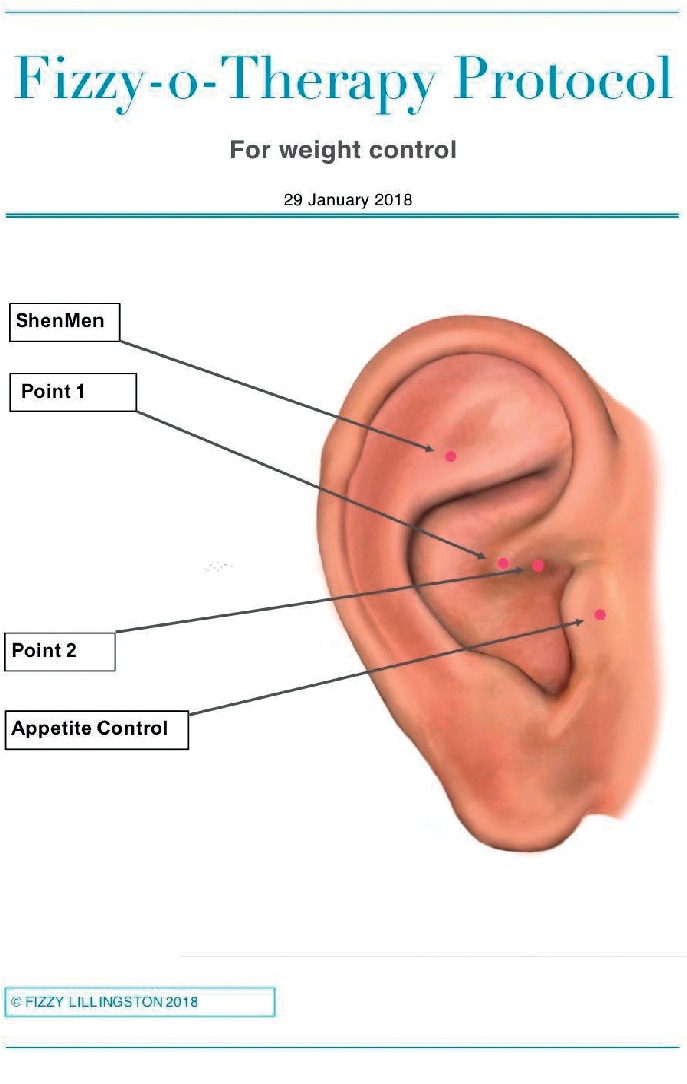
Four-point ear acupuncture weight loss points designed for the trial.

The AA treatment consisted of using sterile press tack needles that had been ordered specifically for the trial. Each pack was checked by the lead researcher to ensure it was sterile and sealed. The same batch of needles was used throughout the trial and new needles were used for every encounter. These 0.2 cm needles were attached to a sterile 1 cm circular piece of wire which was attached to a small plaster so they could stay in place easily for 20 minutes and anyone seeing them could not ascertain if there was a needle under the plaster or not. The four specific acupuncture points used during each treatment are shown in Figure 1.

The sham treatment consisted of a similar plaster as the AA group; however, there was no needle attached. None of the participants had received AA before, and therefore the perceived feeling was unknown to them. The fine needle used in AA is often not felt during a treatment, so differentiating between the sham and the real needles is difficult to ascertain by inexperienced participants. The needles and sham needles were not left in during the week to ensure the participants would not be able to determine which group they had been assigned to.

Data were collected on participant weight, waist circumference, and BMI at the beginning of the study and at one-week intervals for seven weeks. It is worth noting the importance of measuring an accurate waist measurement. A waist measurement is a better predictor of death and chronic disease than measuring just BMI, especially in women [7, 8].

**Figure 2:**
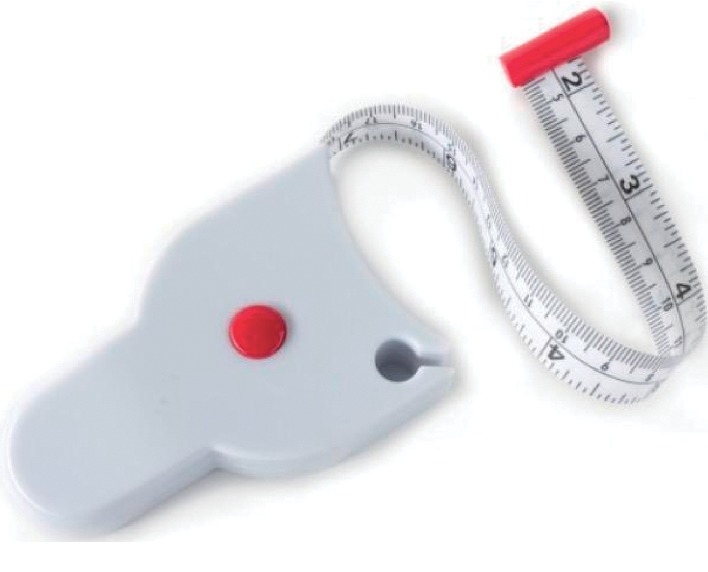
Accurate waist measuring tape.

At the initial consultation and at every appointment, the same electronic weight scale was calibrated and used to avoid error or data collection bias. The tape was designed especially for measuring waist circumference with a secure locking attachment and a button to tighten to the correct size to avoid any error or bias by the person collecting the data (see Figure 2). To ensure unbiased readings were taken, these measures were recorded separately each week. These were stored and entered onto a spreadsheet and analyzed at the end of the study. Computer records of BMI and girth measurement were stored on a password-secured electronic device using a randomly assigned identifying number for anonymity.

Strict criteria were used to measure waist circumference accurately. The measuring tape was placed in a horizontal plane around the abdomen at the level of the iliac crest when standing. The tape was tightened until firm but not compressing the skin and was held parallel to the floor. To achieve this, the loose end was fixed in the receiver and the red button pressed to tighten until firm. The measurement was made at the end of a normal breath expiration. The participants' initial readings for weight, waist circumference, and BMI were treated as covariates in the final data analysis. In addition, each participant's mood, dietary choices, and exercise choices were recorded and also included as covariates. Mood was measured by a Mood and Feelings questionnaire designed by Arnold and Costello [28] and by each subject's weekly self-assessment asking them to rate on a scale of 1 to 5 how happy they felt that day, where 5 was happy and 1 was unhappy or sad.

No dietary advice or intervention was given at any point before or during the study. Any changes in dietary habits could then be attributed to the intervention and not from any advice given.

## 4. Procedure

Upon signing the consent form, each participant was randomly assigned to either the placebo or AA group by the roll of a dice. At weekly intervals for six weeks, the participants, dependent on which group they had been assigned to, were either given the four-point AA treatment especially designed for the trial or sham plasters with no needle, in the same areas. These were left in place for 20 minutes, measured by a timer, and removed immediately following the time expiration. During each weekly treatment, the participants were asked to fill in a weekly record of their mood/feelings together with a food and activities diary. These were issued separately every week to avoid copying over from the week before. All the data were stored electronically at the end of every session in a password-protected database. The data are available for examination by contacting the authors.

Following the six weeks of treatments, the participants were seen one final time during the seventh week for data collection only and not given a treatment. At this point, the participants were told which group they had been assigned to. The placebo group was then offered six further sessions of real acupuncture treatments. A total of 24 of the 28 placebo group participants opted for the complimentary treatment sessions.

## 5. Results

The treatment effect was calculated as the difference in average change when comparing the two groups' changes in weight, BMI, and waist circumference.

### 5.1. Change in Weight

**Figure 3:**
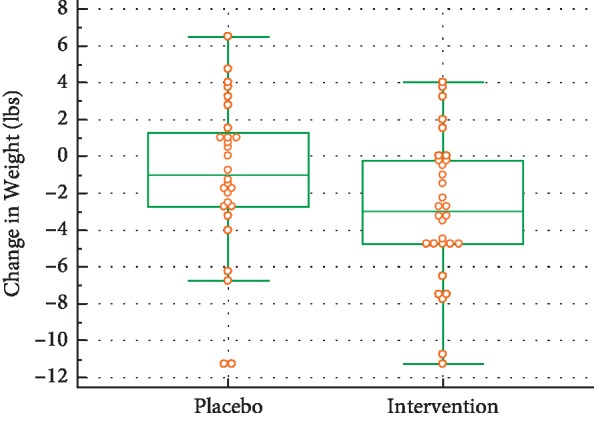
Weight changes during the trial.

The treatment effect was an average change in weight of −1.81 lbs for the AA group compared with the placebo group, which represents a medium effect size (*d* = 0.47) that was only marginally significant (*p*=0.079) (see Figure 3).

### 5.2. Change in BMI

**Figure 4:**
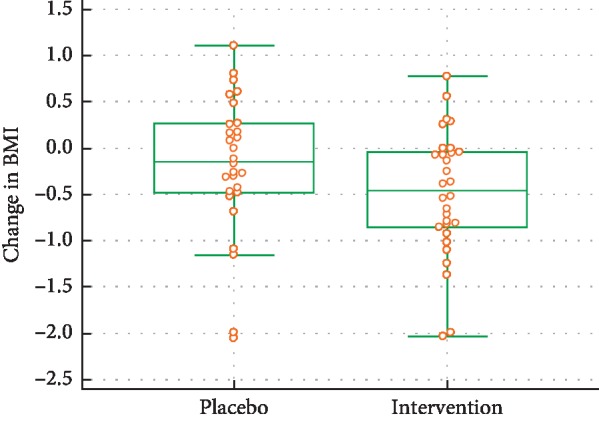
BMI changes during the trial.

The treatment effect was a difference of an average change in BMI of −0.31, which represents a medium effect size (*d* = 0.46) that was only marginally significant (*p*=0.089) (see Figure 4).

### 5.3. Change in Waist Circumference

**Figure 5:**
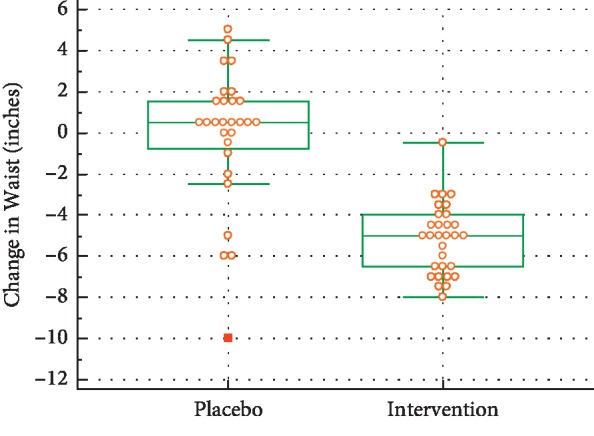
Waist changes during the trial.

There was evidence of a very large and highly significant treatment effect for change in waist circumference, which averaged −4.64 inches (*d* = 1.78, *p* < 0.001) for the AA group compared with the placebo group. However, initially the median waist circumference for the treatment group was 95.0 inches, while for the placebo group it was 87.5 inches. The difference in initial waist circumferences was significant (*d* = 0.72, *p*=0.010). The larger a subject's initial waist circumference, the greater the average decrease in waist circumference related to the treatment (−0.23 inches, *d* = 0.85, *p*=0.002) (see Figure 5).

The *p* value for change-in-waist was highly significant, indicating that the change in waist for the intervention group was different from the placebo group and that the median difference was negative. Interestingly, every single individual in the intervention group experienced a reduction in waist measurement, demonstrating reduced abdominal adiposity, whereas waist measurement results for the control/placebo group were more variable.

### 5.4. Change in Mood

**Figure 6:**
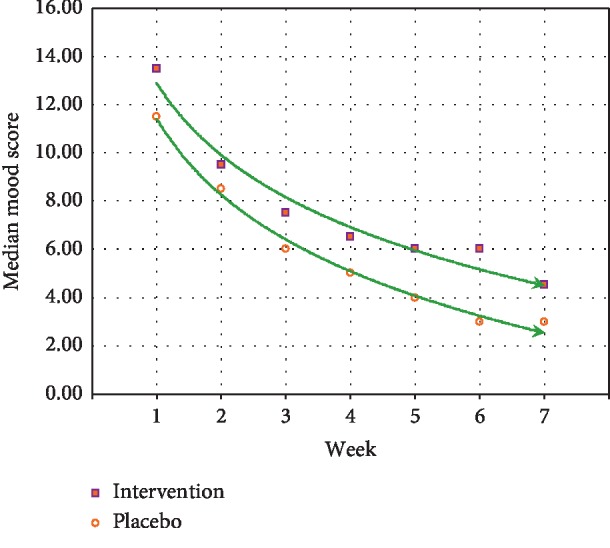
Mood score during the trial.

There was no evidence of a treatment effect on mood (*p*=0.82), but mood improved for both the AA group and the placebo group, and the median improvement in mood (−5.50) was identical for the two groups (see Figure 6). The influence of subjects' initial mood was very large and highly significant (*d* = 2.48, *p* < 0.001) (see Figure 6).

### 5.5. Change in Food Cravings for Sweet and Salty Foods

**Figure 7:**
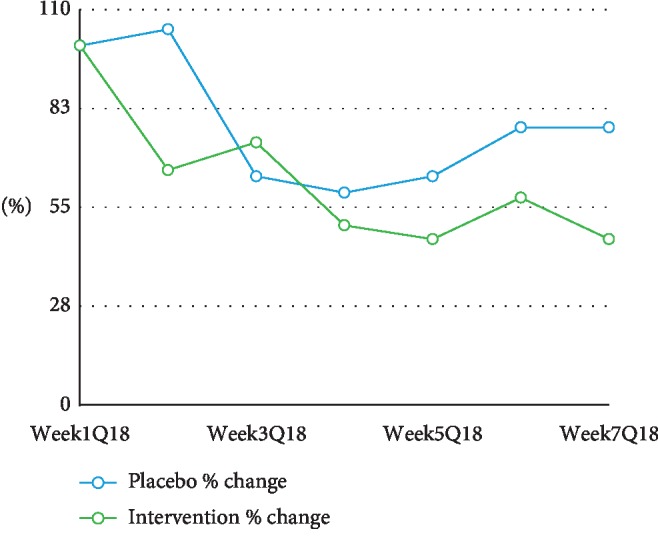
Food cravings for sugar and salt during the trial.

There was a trend noted in reduction in both groups for sweets and salty foods throughout the study, and the intervention group showed a slightly more marked decrease in these cravings (see Figure 7). However, this change was not statistically significant.

## 6. Discussion

Over the six-week duration of the intervention, waist circumference was significantly reduced in the AA group compared with the placebo/sham acupuncture control group. Furthermore, 100% of individuals in the intervention group showed a reduction in waist measurement, and by extension, abdominal adiposity, whereas reduction in waist measurement among the control/placebo group was variable. These results are potentially impactful for participant health as weight reduction from the abdomen may be particularly predictive of improved health outcomes given that visceral, adipose fat stored around the abdomen is particularly predictive of poor health outcomes [29, 30]. Abdominal adiposity and a larger waist circumference is more strongly associated with adverse health risks [31] and subsequent mortality, including myocardial infarction, the leading cause of death worldwide, together with multiple chronic diseases including type II diabetes [32]. This effect seems especially true of women, and research demonstrates that the waist is a better predictor of death and chronic disease than measuring just BMI [29]. It is worth noting that other types of fat, especially gluteofemoral fat, may have a protective effect [33]. It is important to define obesity based on the anatomical location of fat rather than on its volume, especially when cardiometabolic risk is considered. Distribution of weight around the abdomen is one of the first areas to be impacted with reduced high-sugar food intake as this visceral fat yields more easily to reduce refined high-sugar diets and exercise [34]. Abdominal fat cells are more biologically active, and a reduction in this fat as opposed to subcutaneous fat has been linked to metabolic improvement [35]. Thus, measuring waist circumference may be a more sensitive measure than weight loss and BMI [29], explaining the significant difference in waist circumference but not in weight and BMI between our groups.

This negative result is not surprising, given that BMI is partially determined by weight, which did not change significantly in the present study. It is possible that a loss in weight and BMI would have been greater if the intervention had run for a longer duration. Future studies could address this by implementing the intervention for 12 or more weeks.

Food and exercise diaries showed similar behaviour for all candidates; however, an interesting trend emerged when examining responses to the questions about cravings for salty or sugary foods. While both groups reported a nonsignificant reduction in cravings for sugary foods, as the study progressed, by the final week of the intervention, cravings among the control group started to rise again while cravings among the AA intervention group remained stable (see Figure 7). While this difference is not significant and cannot explain the reduced waist circumference in the AA intervention group, it could be an important area of inquiry in future longer duration studies as previous evidence indicates that serotonin, opioids, and amino acids including GABA are implicated in the modulation of dopamine release during acupuncture, thus reducing the rush and subsequent increased craving for sugar [36]. It is possible that overweight and obese individuals may have an impairment in the dopamine regulation pathway that moderates reward, motivation, self-control, and interoceptive awareness [37].

The mood and feeling data suggest that participants in both groups showed a statistically significant improvement in mood as the study progressed. This improvement in mood might be attributable to the Hawthorne effect, that is, the change in the subjects' moods was due in part to being studied [38, 39]. The same researcher interacted with both the AA group and the placebo group. The interaction of the researcher with the participants may have produced a positive change in mood that was similar regardless of being a member of the AA intervention or sham control group. Future studies should be designed to control for the interaction of the participants and the interventionist to determine whether AA alone can lead to reduced waist circumference or if improvement in mood must be in combination with AA to initiate waist circumference reduction.

## 7. Limitations

There are several limitations to the present study. One potential confounding element is the higher average waist circumference in the AA intervention versus the control group at the start of the study. Since the intervention group had more circumference to lose, a greater reduction from pre- to postintervention was seen. This is not a likely explanation for the effect however as previous research has indicated that it is more difficult to lose adiposity from around the waist the greater the circumference of the waist. Japanese and British researchers discovered that the more fat the person has, the more sLR11 is produced inhibiting the rate at which calories can be burnt, thereby preventing weight loss. Whittle suggested that the thermogenesis in adipose tissue is correlated with BMI in humans and may explain why at a cellular level, the stored fat is resisting efforts to lose weight [40]. This may explain why those who carry too much weight find it incredibly hard to lose it. A further study found that those who were considered obese as opposed to overweight had a difference in the physiological mechanisms that notify the brain that the person is full and to stop eating when they consumed too many sugary fatty foods [41]. This makes it more difficult to control appetite, and therefore weight loss is more difficult. However, in the present study, those who were more severely obese were able to reduce their waist circumference and lose weight irrespective of the fact that this group of candidates may have found it harder to lose weight. Thus, the intervention group results are even more surprising and potentially significant. Future studies could use semirandomization to ensure that the two groups were equal in mean weight and waist measurement at the start of the intervention.

Another possible confounding element was the length of the trial. A longer intervention might result in a significant reduction in weight as well as waist circumference as the intervention is given more time to reduce cravings and food intake. Given the lack of significant change in cravings or food intake or difference between the AA intervention and control groups in food intake, it is impossible to conclude whether the reduction in waist circumference among the AA intervention group was driven by reduced cravings and food intake. Future studies will need to accurately measure type and quantity of food intake to address this question.

A final potential confounding element was the change in mood from pre- to postintervention for participants in both groups. This increase in mood may be explained by the Hawthorne Effect or the positive impact of the healthcare practitioner-patient relationship. Given this increase in mood across both groups, it is not possible to conclude whether the AA alone was sufficient to drive the decrease in waist circumference or whether the AA in addition to the positive health-care provider-patient relationship and consequent improvement in patient mood is required to drive the reduction in waist circumference. Further studies that control these two variables are needed to answer this question.

## 8. Conclusion

The results of the study provide evidence that among 20–30-year-old overweight females, a treatment of six weekly sessions of auricular acupuncture (AA) is associated with a reduction in waist circumference compared with sham acupuncture.

## Data Availability

The computer-stored data of all the results used to support the findings of this study are available from the corresponding author upon request. Please email rwaechte@sgu.edu at St. George's University with any request. There is no restriction on data access.
